# Harmful female footwear: A public health perspective

**DOI:** 10.1016/j.heliyon.2023.e21297

**Published:** 2023-10-21

**Authors:** Jacek Lorkowski, Mieczyslaw Pokorski

**Affiliations:** a Department of Orthopedics, Traumatology, and Sports Medicine, Central Clinical Hospital of the Ministry of Internal Affairs and Administration, 137 Woloska Street, 02-507, Warsaw, Poland; b Institute of Health Sciences, Opole University, 68 Katowicka Street, 45-060, Opole, Poland

**Keywords:** Footwear, Feet, Stiletto heels, Public health, Woman

## Abstract

Footwear fashion is an instance of a socially formed attitude affecting somatic population health. High-heeled, particularly pointy-toed shoes are posed to structurally distort and overload feet leading to musculoskeletal sequelae. Here we compiled multilanguage website images presenting female footwear produced by the top manufacturers to assess the advertising effects on the prevailing height of heels worn by women. The method was based on the analysis of websites using the command “woman shoes” in scores of languages of the Internet Google browser. We then compared the results of the internet search with those of a live street surveillance of the footwear worn by 100 adult women in the downtown Warsaw metropolis in Poland. We found that stiletto heels with pointed shoe tips significantly predominated in images representing the countries belonging to the Western cultural sphere compared to less affluent world areas where low or flat heels prevailed. However, we noted a gradual departure from the fashion of high heels over the last decade, confirmed by live street surveillance, liable to reflect changes in the website presentations of top shoe manufacturers consistent with increasing awareness of potential harm by high heels. Yet the female aptitude for wearing more physiologic shoe models appears to exceed that resulting from marketing campaigns. Doing away with high-heeled pointy-toed shoes requires intensification of pro-health preventive measures in the field of public health.

## 1 Introduction

In 1920, Charles-Edward Amory Winslow of Yale University, a formative American figure in public health, defined public health as dealing with the whole of a population [1]. The current definition proposed by the WHO defines public health as an organized social effort to ward off disease and improve, promote, protect, or restore health [2]. It is a highly multidisciplinary field influenced by the individual (36 %) and social demeanors (24 %), genetic determinants (22 %), healthcare (11 %), and environmental factors (7 %) [3]. The kind of footwear worn by a person, often influenced by an existing fashion or ‘dress code’, is an instance of the social attitude that may have a somatic health impact [4]. A case in point is an improperly designed shape of female footwear. The two main potentially harmful elements here are high heels and pointy-toed shoes. High-heeled shoes, undoubtedly, emphasize the attractive slenderness of a female figure, a sociocultural-rooted custom shaped over the centuries whereas pointed shoes are more of a temporary marketing gimmick of manufacturers. There has been increasing knowledge that frequent or prolonged use of high-heeled shoes leads to structural foot distortions such as claw toes, bunions, and calluses [5]. Achilles tendon shortens leading to a painful increase in intra-tendinous pressure and tendonitis over time [6]. Further, preferred walking speed and stride length decrease with increasing heel height, which overloads the plantar and calf muscle-tendon-fascia system leading to cramps and gait destabilization, which may distort the vertical musculoskeletal axis of the human body and neuromechanics of walking [7]. Pointed shoes, on the other side, lead to hammertoes, metatarsalgia, and an inflammation of nerves between the toes eventually forming neuroma that may require surgical treatment [8]. The riskiest appears as a combination of high-heeled and pointy-toed shoes, which all too often seem to go in tandem, since the harmful effects multiplicate in speed and intensity.

This study aims to get insights into the inter-dependent aspects of changes in the attitude toward wearing high-heeled pointy-toed shoes reflecting the awareness of the delayed health consequences on the part of both footwear woman users and manufacturers. These aspects included the country's affluence making it easier to adopt new fashion trends and the extent to which knowledge of foot deformity-related neuromuscular dysfunction could affect manufacturers' ways of advertising female footwear, an influential fashion trendsetter at the population level. We acted on the premise that a quantitative determinant of the prevalence of a social phenomenon is the frequency of its occurrence noted on the website. To this end, we analyzed multilanguage compilations of shoe-presenting website images. Additionally, we performed a street surveillance of the type of shoes worn by adult women in the downtown Warsaw metropolis in Poland assessing the implicit effects of web advertising.

## 2 Subjects and methods

This study was performed in Warsaw, Poland, in October 2021. The study protocol consisted of three stages. We used the internet Google browser to perform an image search using the command “woman shoes”, considered the simplest and most used by an average internet user interested in the subject. First, we analyzed the images that contained a recognizable type of footwear, irrespective of brand name shoe manufacturers, which were presented in the following 30 languages representing all continents: English, German, French, Spanish, Portuguese, Polish, Dutch, and Basque; Hebrew, Greek, Turkish, Japanese, Ukrainian, Russian, Arabic, Chinese, Hindi, Bengali, Korean, Nepali, Somali, Vietnamese, Malay, Mongolian, Kazakh, Kyrgyz, Afrikaans, Indonesian, Zulu, and Swahili. The first eight languages listed were singled out to form a separate comparative group. The rationale for highlighting these languages was that they were strongly related to the Western world civilization sphere according to Huntington's division [9]. As such, these languages were thought to represent societies that have social and cultural adroitness at adapting fashion trends, including footwear. The first ten images appearing in each language with recognizable types of footwear were considered, based on Google algorithms indicating that the first appearing internet images are most frequently called upon with a given search command and thus are ranked as being of higher relevance [10]. The search was carried out after the computer had been cleared of browsing history and any preferences.

Second, using the search commands “company name” AND “woman shoes” AND 2021 and then “company name” AND “woman shoes” AND 2011, we compared the 2021 and 2011 shoe image presentations of 14 reputable international manufacturers such as Calvin Klein, Venezia, Bugatti, Kazar, Baldmini, Manolo, Blahnik, Bottega, Hegos, Lilu, Jimmy Cho, Louboutin, Saint Laurent, and Versace. This search was limited to the English language only. Again, the first 10 images of each company were considered.

Third, we performed live street surveillance of the type of footwear worn by 100 adult anonymous women aged 20–50, consecutive mid-day passers-by in the Warsaw downtown area on the sunny afternoon of a working day between 5–6 p.m.

For the analysis, we considered the following categories of shoes reflecting the influence on the locomotor system:

• Category 1: stiletto heels, >10 cm, with pointed and non-pointed tips;
• Category 2: moderately high heels, 4–10 cm, with pointed and non-pointed tips and flat shoes with pointed tips;
• Category 3: low <4 cm or flat heels with non-pointed tips and sports shoes such as sneakers, athletic shoes, tennis shoes, gym shoes, running shoes, and sandals.

Quantitative data were presented as means ± SD. Data were compared using the Kruskal-Wallis H test with post-hoc Dunn's test for within-group differences and the Mann-Whitney *U* test was used for comparisons of intergroup differences between the corresponding categories of heel height. A p < 0.05 defined a statistically significant difference.

## 3 Results

### 3.1 Heel height depending on the country language tested

There were significant differences in the number of shoe images representing the three categories of heel height in both Western (p = 0.029) and other languages (p = 0.00006) investigated (Table 1). Pairwise comparisons of the corresponding categories showed a significant preponderance of stiletto heels (Category 1) over high heels (Category 2) in Western languages (p = 0.009) and low heels, flats, and sports shoes (Category 3) over high heels (p = 0.00003) and stiletto heels (p = 0.001) in other languages.

**Table 1:** Number of shoe images in each category of heel height by the language group.

Heel height	Languages	
	Group I (8 Westerns)	Group II (22 others)
Category 1 (>10 cm)	4.88 ± 1.90^††^	2.14 ± 2.60
Category 2 (4–10 cm)	2.13 ± 1.90*	1.05 ± 1.19
Category 3 (<4 cm)	2.88 ± 1.36^†^	5.50 ± 3.19**

Data are means ± SD. *p < 0.010 and **p < 0.001 different from Category 1 in respective language groups; †p < 0.030 between corresponding Categories 3 and ††p < 0.010 between Categories 1.

Further, a breakdown of stiletto heels (Category 1) into the pointed vs. non-pointed tips showed significant numerical superiority of the former – 3.75 ± 1.30 vs. 1.12 ± 0.93 (p < 0.001) – in Western languages. The two types of shoe tips were about evenly distributed in other languages – pointed 1.00 ± 1.38 vs. non-pointed tips 1.14 ± 1.52. In contrast, high heels in Category 2 showed remarkable numerical superiority of non-pointed tips in both Western, 0.00 ± 0.00 vs. 1.38 ± 1.11 (p < 0.006) and other languages, 0.00 ± 0.00 vs. 0.82 ± 1.27 (p < 0.005).

### 3.2 Heel height depending on the position of brand name manufacturers over the past decade

The number of shoe images in each category of heel height on the websites of top world shoe manufacturers, comparing 2021 vs. 2011 is shown in Table 2. There were differences among the three categories of heels in either year, with images of stiletto heels (Category 1) significantly outnumbering the remaining two categories in both years. However, differences in the image number between the corresponding heel-height categories remained insignificant over a decade apart.

**Table 2:** Number of shoe images in each category of heel height considering position change of brand name manufacturers (n = 14) over the past decade.

Heel height	Year	
	2021	2011
Category 1 (>10 cm)	7.29 ± 2.58*	6.43 ± 3.62^†≠^
Category 2 (4–10 cm)	1.14 ± 1.51	0.93 ± 1.75
Category 3 (<4 cm)	1.57 ± 1.76	2.64 ± 3.13

Data are means ± SD. *p < 0.0001 different from Category 2 and 3 in 2021; ^†^p = 0.0001 different from Category 2, and ^≠^p = 0.025 different from Category 3 in 2011.

A breakdown of stiletto heels showed a significant preponderance in the number of images with pointed over non-pointed tips – 5.14 ± 2.90 vs. 2.14 ± 1.36 (p = 0.0004) in 2021 and 5.43 ± 3.68 vs. 1.00 ± 1.36 (p = 0.0023) in 2011. Concerning the high heels (Category 2), the distribution of pointed and non-pointed tips was about equal in the respective years: 0.50 ± 0.73 vs. 0.57 ± 1.12 (p = 0.424) and 0.36 ± 1.29 vs. 0.29 ± 0.76 (p = 0.866).

### 3.3 Live street surveillance of Women's footwear

**Fig. 1:**
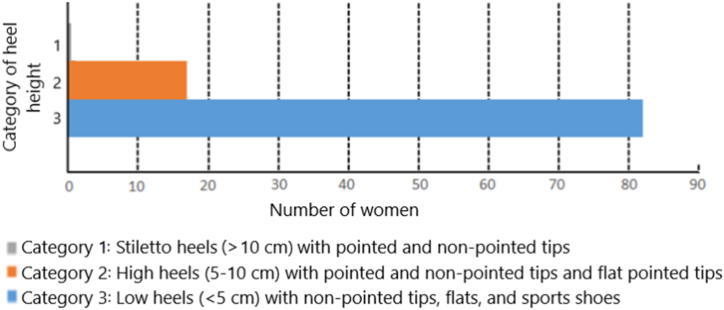
Count of women wearing shoes in the respective heel-height category during live street surveillance.

Finally, in live street surveillance of the type of footwear worn by 100 adult women, we found that none wore stiletto heels either with or without pointed tips (Category 1). Eighteen women wore shoes with high heels of 5–10 cm with pointed and non-pointed tips, or flats with pointed tips (Category 2), and 82 women wore low heels (<5 cm) with non-pointed tips or various sports shoes (Category 3). These results are depicted in Fig. 1.

## 4 Discussion

This study focused on the prevalence of wearing high-heeled shoes by contemporary women as influenced by website advertising of shoe manufacturers at the time of increasing awareness of the harmful impact on foot health. We used the Internet Google browser to search for images of footwear in scores of languages. The rationale was that the web is influential in advertising and selling brand name shoes and thus in shaping female culture and habit of choosing footwear fashion and acquiring shoes. We also assessed how the web displays of top shoe manufacturers adapted to increasing knowledge of the potentially harmful impact of high heels over a decade time. Finally, we applied the collected web observations to a live surveillance of shoes worn by women in the downtown Warsaw metropolis in Poland.

We found a distinguishable difference in the predominating fashion of shoes displayed in images representing the countries belonging to the Western cultural sphere compared to less affluent world areas. In the former group, stiletto heels significantly predominated, with a notable advantage of pointy-toed shoe tips, whereas in the latter, low or flat-heeled shoes predominated over the stiletto and high-heel categories. The number of shoe images in the high heel category representing the non-Western group was less than half of that in the Western language group. Moreover, the Western predominance of shoes with pointed tips was no longer observable.

We further noted a gradual departure from the fashion of high heels, particularly observable in the last decade in Western countries whose female inhabitants were more prone to adopt new fashion trends. That trend was reflected in the shifting way, the shoe brands were presented on the manufacturers’ websites over the past decade. These presentations tilted toward flat heels and a variety of sports footwear, shying away from stiletto heels. The change was confirmed by a live street observation of the heavy predominance of women wearing flat heels or sports shoes in the downtown Warsaw metropolis in Poland.

Biomechanically, the foot is the final element in the kinetic chain of the human body. The foot conforms to the upright posture through longitudinal and transverse arches [11]. During walking on high heels, dorsiflexion is significantly reduced in the metatarsophalangeal joints, which adversely affects the transverse arch [12]. Particularly, covered pointed tips exert increased pressure within toes and metatarsophalangeal joints leading to foot deformation [13,14]. Such changes, even when small in magnitude, change body contours and disrupt the natural biomechanics of the foot, leading to the development of hallux valgus, calluses, transverse flatfoot, osteoarthritis, and overload syndrome [[15], [16], [17], [18]]. Changes in the axial skeleton take hold of the entire torso including respiratory and lower limb muscles and shift the center of gravity forward, which distorts gait kinematics and enhances energy expenditure to maintain the body balance [19,20]. The severity of degenerative pathologies is determined by the cumulative time of wearing high heels, body height and mass, foot morphology, and decreasing compensatory ability with age [21]. Foot disorders increase overall morbidity and quality of life [22,23].

High heels are an ancient story, with mural illustrations in Egypt dating back to 3500 BCE [24]. Female foot restraining to obtain a specific, non-physiological shape was common practice in high call women in ancient China and Japan as well. This custom stemmed from the millennia-old tradition conditioned by fashion and a model of beauty adopted by large population segments. A similar type of shoe appeared in the Middle Ages, emulating the ancient Greek wedges [25]. However, their purpose was different as they were meant to keep the feet as much above the medieval street dirt as possible. Wedges were worn mainly by men at the time, and they offered a more evenly distributed pressure throughout the foot and better arch support conferring less distortion of lower limb joints and the backbone compared to contemporary high heels [17]. In Europe, the origin of high heels is most associated with Catherine de Medici and Louis XIV in the XVI and XVII Centuries. The use of this non-physiological footwear originally restricted to the nobility was later increasingly extended to the general population as a fashionable expression of female beauty, along with technological advances in shoe manufacturing [26]. In modern times, a proportion of women still choose to wear high heels, still considered by some as a symbol of feminine sexuality [27,28]. Wearing high heels increases the attractiveness of women in the eyes of men. High heels also confer a psychosexual reward, strengthening women's self-acceptance [29,30]. Wearing such shoes is influenced by advertising, media, and the celebrity lifestyle [31,32]. Studies show that more than two-thirds of women wear high-heeled ill-fitted shoes not because of their free choice but to comply with unwritten social pressure and norms [33], no matter how irrational that might be from the health standpoint. The canons of beauty mitigate the scope of women's freedom but fulfill public expectations.

According to the literature, 59 % of corporate women in industrialized countries wear high heels for up to 8 h a day [22]. Seventy percent of them report a deterioration of their quality of life due to periodic bouts of foot pain and reduced mobility, which becomes a serious public health issue. The scale of the problem may well be underestimated and takes on the size of the epidemics of the XXI Century [17,34]. Research shows that the higher the heel, the more misaligned the foot is. Therefore, high heels should be shunned, used for the shortest possible time on special occasions only, and taken off straight afterward. High and pointy shoes exacerbate the harm by cramping the toes in unnatural shape leading to distorted musculoskeletal geometry and local neuroinflammatory sequelae. High-heeled or pointed shoes must not be worn until the female skeleton reaches full maturity [27,35,36]. Despite the knowledge of harmful health effects, adolescents are particularly susceptible to peer and advertising pressures, which may factor in their frequent wearing of such shoes [20,28,37]. Wearing a low-heeled variety with strong heel counter support and a wide enough toe box should be prioritized.

This study has limitations linked to possible biases inherent in designs containing subjective elements such as the wording of internet search commands, grouping of languages for internet search, and search algorithm manipulations. There is yet no perfect way of collecting data in Google search enabling a user to have full-scale insights about visitors on the site. We chose to rely upon the first ten images appearing, based on the common algorithm that the higher the web positioning of a product at the top places, the more attention it garners and the more effective its promotion. Sufficiency of this seemingly small number of images for analysis is supported by the knowledge that data collected have an optimal small bounce rate, i.e., the percentage of users who left the site straight after the appearance of a landing page, and a good conversion rate, i.e., the percentage of users who acquired the advertised item from among the images opened. Increasing the number of images does not optimize the search from a holistic perspective [9,38]. Nonetheless, our findings reflect the specific study design employed and may not carry the air of ubiquitousness.

## 5 Conclusions

This study shows that high heels, carrying the bone-muscular degenerative potential for the entire foot-spine axis and particularly for foot health, remain the height of fashion in website images in Western languages representing upscale countries whose female inhabitants are most prone to adopting and consuming new fashion trends. Such shoes, however, appear significantly less fashionable among women of less affluent societies at the expense of low-heeled shoes, flats, and sports shoes. The increasing awareness and knowledge of the harmful effects of wearing high heels likely stand behind the signs of a shift over the past decade toward images focusing on low heels, flats, and sports shoes while gradually shunning away from high heels in website marketing campaigns of footwear manufacturers. The shift of paradigm concerning high heels was confirmed in the present study by live street surveillance of the footwear worn by women in the bustling environment of the Warsaw metropolis in Poland, a country included in the Western sphere group in this study. However, the social trend for wearing more physiologic shoe models appeared to exceed that resulting from marketing campaigns. Doing away with high heels requires intensification of pro-health measures in the field of public health based on evidence-based podiatric research.

## Ethical approval

Procedures performed in this study were in accord with the 1964 Helsinki Declaration and its later amendments, national legislation, and institutional requirements. This study did not involve any direct contact with individuals. Therefore, ethics and consent-seeking requirements were waived by the institutional research review committee.

## Data availability statement

The authors confirm that the data supporting the findings of this study are available within the article. The raw data are deposited with the author JL and are available upon reasonable request.

## CRediT authorship contribution statement

**Jacek Lorkowski:** Conceptualization, Data curation, Formal analysis, Investigation, Methodology, Validation, Writing – original draft. **Mieczyslaw Pokorski:** Conceptualization, Data curation, Formal analysis, Investigation, Methodology, Writing – review & editing, Validation.

## Declaration of competing interest

The authors declare that they have no known competing financial interests or personal relationships that could have appeared to influence the work reported in this paper.
